# Invasive potential of *Borrelia burgdorferi* sensu stricto *ospC* type L strains increases the possible disease risk to humans in the regions of their distribution

**DOI:** 10.1186/s13071-014-0538-y

**Published:** 2014-11-28

**Authors:** Maryna Golovchenko, Radek Sima, Ondrej Hajdusek, Libor Grubhoffer, James H Oliver, Nataliia Rudenko

**Affiliations:** Biology Centre AS CR, Institute of Parasitology, Ceske Budejovice, 37005 Czech Republic; Georgia Southern University, James H. Oliver, Jr. Institute of Coastal Plain Sciences, Statesboro, GA 30460-8056 USA; University of South Bohemia, Ceske Budejovice, 37005 Czech Republic

**Keywords:** *B. burgdorferi ospC* type, Invasive potential, Lyme disease, Southeastern U.S.A., Tick vector, Vertebrate host

## Abstract

**Background:**

Analysis of *Borrelia burgdorferi ospC* types from the southeastern U.S.A. supported the common belief that various *ospC* types are geographically restricted and host specific. Being widely distributed in the region, the southeastern population of *B. burgdorferi* is represented by a surprisingly small number of *ospC* types. Types B, G and H are dominant or common and are invasive, while scarce type L, restricted mostly to the southeastern U.S.A., is believed to rarely if ever cause human Lyme disease. *OspC* type B and L strains are represented in the region at the same rate, however their distribution among tick vectors and vertebrate hosts is unequal.

**Findings:**

Direct diagnostics was used to analyze the ability of *B. burgdorferi ospC* type L strains to disseminate into host tissues. Mice were infected by subcutaneous injections of *B. burgdorferi* strains of various *ospC* types with different invasive capability. Spirochete levels were examined in ear, heart, bladder and joint tissues. Noninfected *I. ricinus* larvae were fed on infected mice until repletion. Infection rates were determined in molted nymphs. Infected nymphs were then fed on naïve mice, and spirochete transmission from infected nymphs to mice was confirmed.

**Conclusions:**

*B. burgdorferi ospC* type L strains from the southeastern U.S.A. have comparable potential to disseminate into host tissues as *ospC* types strains commonly associated with human Lyme disease in endemic European and North American regions. We found no difference in the invasive ability of *ospC* type B and L strains originated either from tick vectors or vertebrate hosts.

## Findings

### Background

It is known that each *Borrelia burgdorferi* sensu lato (s.l.) species is characterized by its tick vectors, host spectrum, geographical distribution and, for the pathogenic species, its organotropism [1]. The relative invasiveness of various *B. burgdorferi* strains, classified by *ospC* type, reveals the ratio between that type‘s frequency in vector ticks compared to human patients [2].

Published data on prevalence of Lyme disease (LD) spirochetes in vector ticks and vertebrate hosts in the southeastern U.S.A. qualifies this region as *B. burgdorferi* s.l. endemic area, despite prevailing dogma concerning LD in the United States [3]. Briefly, current dogma states that *Ixodes scapularis* (formerly, *I. dammini*) is the only vector of the spirochetes in the eastern U.S.; *B. burgdorferi* is antigenically and genetically uniform in North America in contrast to the situation in Europe; *B. burgdorferi* does not occur in wildlife in the southern U.S.A. and thus, humans in the Southeast could not acquire LD [3,4]. It is now known that the often referenced *I. scapularis-Peromyscus leucopus* enzootic cycle in the northeastern U.S.A. is mirrored by the *I. scapularis-P. gossypinus* transmission cycle in the Southeast which is further enhanced by *Ixodes affinis* and *Ixodes minor* as vital maintenance vectors of *Borrelia* in areas where they occur, and *Sigmodon hispidus* and *Neotoma floridana* as additional major reservoir hosts [3,5]. As the tick vectors and reservoir hosts differ significantly between the northeastern and southeastern regions, it is highly possible that *B. burgdorferi* strains that cause LD in both areas will differ as well. Our previous research showed that of the 4 *ospC* types, B, G, H and N that have been detected in LD patients in the northeastern and midwestern U.S.A., 3 types, B, G, and H, at a lower rate, are widely distributed in the southeastern United States [5]. While *OspC* type B is associated with severe LD around the world [6,7], *ospC* types H and G are commonly detected in tissues at disseminated sites of LD patients from the northeastern and midwestern U.S.A. [2,8]. *B. burgdorferi ospC* type L strains were not considered to have any impact in LD in North America in general and, in the southeastern U.S.A. particularly, because: i) *ospC* type L was considered to be restricted to Europe only; ii) *ospC* type L is recognized as very rare worldwide; and iii) *ospC* type L strains were previously detected in ticks only [2,6–9].

Globally rare *B. burgdorferi ospC* type L strains are associated with the non-human biting tick *I. affinis* that feeds on the same vertebrate hosts as human-biting *I. scapularis* in the southeastern U.S.A. [9]. However, due to the common reservoir hosts in the feeding cycle, *Borrelia* strains from *I. affinis* might be transmitted to humans [3]. *B. burgdorferi ospC* types B and L strains are the most prevalent in the region. They are represented at the same rate overall, but their distribution among the tick vectors and the rodent hosts is unequal. While 75% of highly invasive *ospC* type B strains were isolated from rodents and 25% from ticks, in the case of *ospC* type L, host- and vector - originated strains represented 37.5% and 62.5%, respectively. For a long time it was believed that *ospC* type L strains are incapable of causing human LD [2]. Nevertheless, a *B. burgdorferi ospC* type L strain (SLV-1) was cultured from a skin biopsy from a Slovenian patient with acrodermatitis chronica atrophicans (ACA), commonly associated with *B. afzelii* infection [10]. Additional strains were isolated from *I. ricinus* nymphs collected in Switzerland (NE5222 and NE5266) and Slovakia (SKT-2) [5], European LD endemic regions. Another *ospC* type L strain (SCW-9) was isolated from secondary sites of infection of cotton rat (*Sigmodon hispidus*) trapped in South Carolina, U.S.A. [5,9]. The question about Lyme disease in the southeastern U.S.A. is still controversial and confounded by multiple facts and fallacies. Because *ospC* type L strains are one of the two most prevalent in this region [9], the goal of this study was to analyze the capability of *B. burgdorferi ospC* type L strains to disseminate into vertebrate hosts and to compare their invasive potential with infective strains.

### Methods

Low passage *Borrelia* strains were grown in BSK-H medium (Sigma-Aldrich, U.S.A.) according to the protocol described previously [11,12]. Strains used for infection of laboratory mice were characterized in our previous study [5] and were selected on the basis of their *ospC* types defined earlier [5]: human originated *ospC* type B strain SLV-2 (collection site 46°3′17″N,14°30′21″E), cotton mouse (*Peromyscus gossypinus*) originated *ospC* type L strain SCCH-30 (32°47′00″N, 79°56′00″W), *I. ricinus* nymphs originated *ospC* type L strain NE5222, *ospC* type B strain NE5264 and *ospC* type V strain NE5248 (46° 59' 34.72″N, 6° 55′ 54.96″E) and *I. affinis* female originated *ospC* type B strain SCW-53 (33°10′17″N, 79°23′57″W). Six weeks old female C3H/HeN mice (Jackson Laboratory, Sulzfeld, Germany) were infected by subcutaneous injections of 10^5^ spirochetes in 100 μl of BSK-H medium per mouse (2 mice per strain). Presence of spirochetes in ear biopsies was determined at weekly intervals by PCR. Total DNA was extracted using a NucleoSpin Tissue Kit (Macherey-Nagel, Germany) according to the manufacturer’s protocol. Detection of spirochetes was performed by amplification of a 154 bp fragment of flagellin gene using primers FlaF1 (AAGCAAATTTAGGTGCTTTCCAA) and FlaR1 (GCAATCATTGCCATTGCAGA) [12]. At the 3^rd^ week after infection the presence of spirochetes was confirmed in all ear biopsy samples regardless of the *ospC* type of strain used for infection. *I. ricinus* larvae were obtained from the pathogen-free tick colony of the Institute of Parasitology (Czech Republic). At the 4^th^ week after inoculation, non-infected larvae were fed on infected mice until repletion (100 larvae per mouse) and left to molt. Infection rates were determined in pools of ten molted nymphs using PCR described above. Nymphs were considered to be infected if >80% of them were PCR positive. Ten infected nymphs were then fed on naïve mice (5 mice per strain); spirochete transmission from infected nymph to mice was determined as described above. At week 6^th^, spirochete load was determined in positive biopsies from mouse ear, heart, bladder and joint by quantitative PCR using primers described above, the TaqMan FlaProbe1 (TGCTACAACCTCATCTGTCATTGTAGCATCTTTTATTTG) and a LightCycler 480 (Roche) [13]. Spirochete burden in tissues was normalized to mouse actin using previously described methods [14]. Amplification and sequencing of spirochete *ospC* genes from final mouse samples confirmed their identity to the *ospC* genes of *B. burgdorferi* strains used for initial infection [5]. All laboratory animals were treated in accordance with the Animal Protection Law of the Czech Republic No. 246/1992 Sb., ethics approval No. 137/2008.

### Results and conclusions

PCR was used to confirm infection of laboratory mice with invasive *B. burgdorferi ospC* type B strains and *ospC* type L strains, previously found only in ticks (Table 1). An *ospC* type V strain was included in the analysis for comparison, as it was found in ticks and in sites of local infection in humans. The same pattern of transmission of *ospC* type B and type L strains of the LD spirochete from infected host to tick vector and then from infected tick vector to uninfected host was revealed using the designed protocol. It is interesting to note that the results of dissemination of host originated *ospC* type B (human, SLV-2) and L (rodent, SCCH-30) strains were comparable in each tissue analyzed (Table 1). Dissemination ability of *ospC* type B (NE5264) and L (NE5222) strains originated from human biting *I. ricinus* was almost equal in each tissue (Table 1). However, the load of spirochetes of *ospC* type B (SCW-53) originated from non-human biting *I. affinis* was more than 10 times higher in mouse joints than was the spirochete load of human-originated *ospC* type B (SLV-2) or human-biting *I. ricinus* originated (NE5264) strains. Our results confirm that tick originated strains NE5264 and NE5222 of *ospC* types B and L and host originated *ospC* type B strain SLV-2, show the same pattern of dissemination in all analyzed host tissues, with no preferential site of infection. Identical pattern of dissemination was revealed for non-human biting tick originated *ospC* type B strain SCW-53, and rodent originated *ospC* type L strain SCCH-30 (strains from South Carolina, U.S.A.), with joints as the preferable site of spirochete accumulation. Analysis of *I. ricinus* originated *ospC* type V strain NE5248 showed that, in contrast to types B and L, this *ospC* type preferably disseminates into bladder, not joints (Table 1).

**Table 1:** **Invasive potential of** ***B. burgdorferi*** **sensu stricto strains with different** ***ospC*** **types**

**Mice**		***Borrelia***					**Ear punch biopsy (week)**						**qPCR results (#)**			
**Qty**	**Strain**	**Origin**	**Species**	**Strain**	**Source**	***ospC***	**1**	**2**	**3**	**4**	**5**	6*	**Ear**	**Bladder**	**Joint**	**Heart**
2	C3H/HeN	Europe	*Bb s.s.*	SLV-2	human	type B	-	-	+	+	+	2 ∕ 2	12.0 ± 3.0	9.5 ± 2.5	15.0 ± 9.0	3.0 ± 1.0
5	C3H/HeN	U.S.A.	*Bb s.s.*	SCCH-30	*P.gossypinus*	type L	-	-	+	+	+	4 ∕ 5	7.0 ± 2.4	7.0 ± 1.2	33.0 ± 18.5	5.0 ± 1.0
2	C3H/HeN	Europe	*Bb s.s.*	NE5264	*I. ricinus* (n)	type B	-	-	+	+	+	2 ∕ 2	18.5 ± 5.3	18.7 ± 9.5	14.6 ± 1.8	2.9 ± 0.4
5	C3H/HeN	Europe	*Bb s.s.*	NE5222	*I. ricinus* (n)	type L	-	-	+	+	+	3 ∕ 5	14.6 ± 2.7	20.7 ± 6.2	14.0 ± 3.4	2.5 ± 0.3
2	C3H/HeN	U.S.A.	*Bb s.s.*	SCW-53	*I. affinis* (f)	type B	-	-	+	+	+	2 ∕ 2	6.5 ± 0.5	12.0 ± 1.0	174.5 ± 70.5	3.0 ± 2.0
2	C3H/HeN	Europe	*Bb s.s.*	NE5248	*I. ricinus* (n)	type V	-	+	+	+	+	1 ∕ 2	8	20	6	4

Qty- quantity: # of mice/experiment; (n) - nymph; (f) - female; 6*- total number of infected mice at 6 weeks time point; qPCR results are expressed as means ± SEM, (#) - number of spirochetes/10^5^ murine genomes.

*B. burgdorferi ospC* type L strains originated from human, rodent or hard tick were able to disseminate into laboratory mice as well as invasive *ospC* type B strains that are responsible for systemic disease in humans. Therefore, further study on the pathogenicity of *B. burgdorferi ospC* type L strains is warranted. The qPCR results in this study defined heart as a tissue with the lowest load of spirochetes, while the joints showed the highest load of borrelia in mice infected either with *ospC* type B or L strains (Table 1). Our results support the association of *B. burgdorferi* with Lyme arthritis [15,16]. The confirmed ability of *ospC* type L strains to disseminate into vertebrate host tissues in the same manner as invasive *ospC* type B strains, known to be responsible for severe disease in humans worldwide, increases the possible disease risk to humans in the southeastern U.S.A., the region, where studied strains are widely distributed [5].
